# Poor usability of computer-assisted navigation for hip fracture surgery

**DOI:** 10.1007/s00402-023-05096-2

**Published:** 2023-10-25

**Authors:** Rasmus Abildtrup Hestehave, Per Hviid Gundtoft, Christian Lind Nielsen, Ole Brink, Jan Duedal Rölfing

**Affiliations:** 1 Department of Orthopaedics, Aarhus University Hospital, Palle Juul-Jensens Boulevard 99, J801, 8200 Aarhus, Denmark; 2 Corporate HR, MidtSim, Central Denmark Region, Hedeager 5, 8200 Aarhus, Denmark

**Keywords:** “Hip fractures” [Mesh], “Femoral fractures” [Mesh], “Fracture fixation, intramedullary” [Mesh], “Surgery, computer-assisted” [Mesh], Stryker, ADAPT, Gamma3, System usability scale, Usability

## Abstract

**Introduction:**

The STRYKER ADAPT computer-assisted navigation system provides intraoperative feedback to the surgeon regarding implant placement of the Gamma3 nail. The usability of the ADAPT system has not been evaluated. The aim of the study was to investigate the perceived usability of the ADAPT system.

**Materials and methods:**

This was a descriptive study with prospectively collected data. ADAPT was introduced at Aarhus University Hospital in February 2021. Prior to introduction, surgeons at the department attended a general introduction to the system. ADAPT was introduced to the surgical nurses and was on display at the surgical ward at more than one occasion, where personal introduction to the system was possible. After introduction, it was mandatory to use ADAPT when using the Gamma3 nail to treat intertrochanteric femur fractures. After each procedure, primary and an eventual supervisor answered a questionnaire, which encompassed the System Usability Scale (SUS) questionnaire. The SUS is a ten-item questionnaire regarding the perceived usability of a system. SUS scores were translated to adjectives, describing user experience on a 7-point adjective scale (worst imaginable, awful, poor, ok, good, excellent, best imaginable). User acceptability, defined as “not acceptable”, “marginal” or “acceptable”, was also used to interpret the SUS scores.

**Results:**

ADAPT was used in 50 procedures by 29 different surgeons, with varying skill-level. Median SUS-score after first-time use of ADAPT for all 29 surgeons was 43 (range: 5–60), which translated to “poor” or “not acceptable”. For surgeons who performed ≥ 3 ADAPT-assisted procedures, there were no statistically significant difference in their first to latest SUS-score (median difference: 4.3, *p = *0.5). In free text comments ADAPT was positively described as helpful in placement of K-wire and providing educational opportunities for inexperienced surgeons and negatively as inconsistent, slow, time consuming, and causing excessive fluoroscopy.

**Conclusions:**

Usability and acceptability of ADAPT was rated as “poor” or “not acceptable” by the majority of operating surgeons. ADAPT has not been used at our institution based on these findings. The System Usability Scale may be used in further research exploring usability and acceptability of novel computer-assisted navigation systems for orthopaedic surgery.

**Supplementary Information:**

The online version contains supplementary material available at 10.1007/s00402-023-05096-2.

## Introduction

Intertrochanteric proximal femoral fracture is a common injury in the elderly population with an estimated incidence rate of 100–150/100.000 person years [1, 2]. Intertrochanteric fractures require surgical intervention with adequate reduction and internal fixation. The optimal implant for intertrochanteric fracture fixation with either an intramedullary nail (IMN) or a sliding hip screw has not yet been clearly established [3–5]. Consequently, the choice of implant depends not only on the fracture classification, but also preference of the surgeon and other factors [6]. Lack of proper reduction and sub-optimal placement of the implant are factors that increases the risk of failure. More specifically, the risk of screw cut-out and re-operation of both sliding hip screws and IMN have been shown to correlate with the tip-apex distance (TAD), which is the sum of the distance from the tip of the lag screw to the apex of the femoral head on AP and lateral views [7, 8]. The technical skills needed for proper implant positioning can be safely trained outside the operating theater [9–12]. Intraoperatively, computer-assisted navigation systems may aid both novice as well as experienced surgeons to achieve this goal [13].

**Fig. 1:**
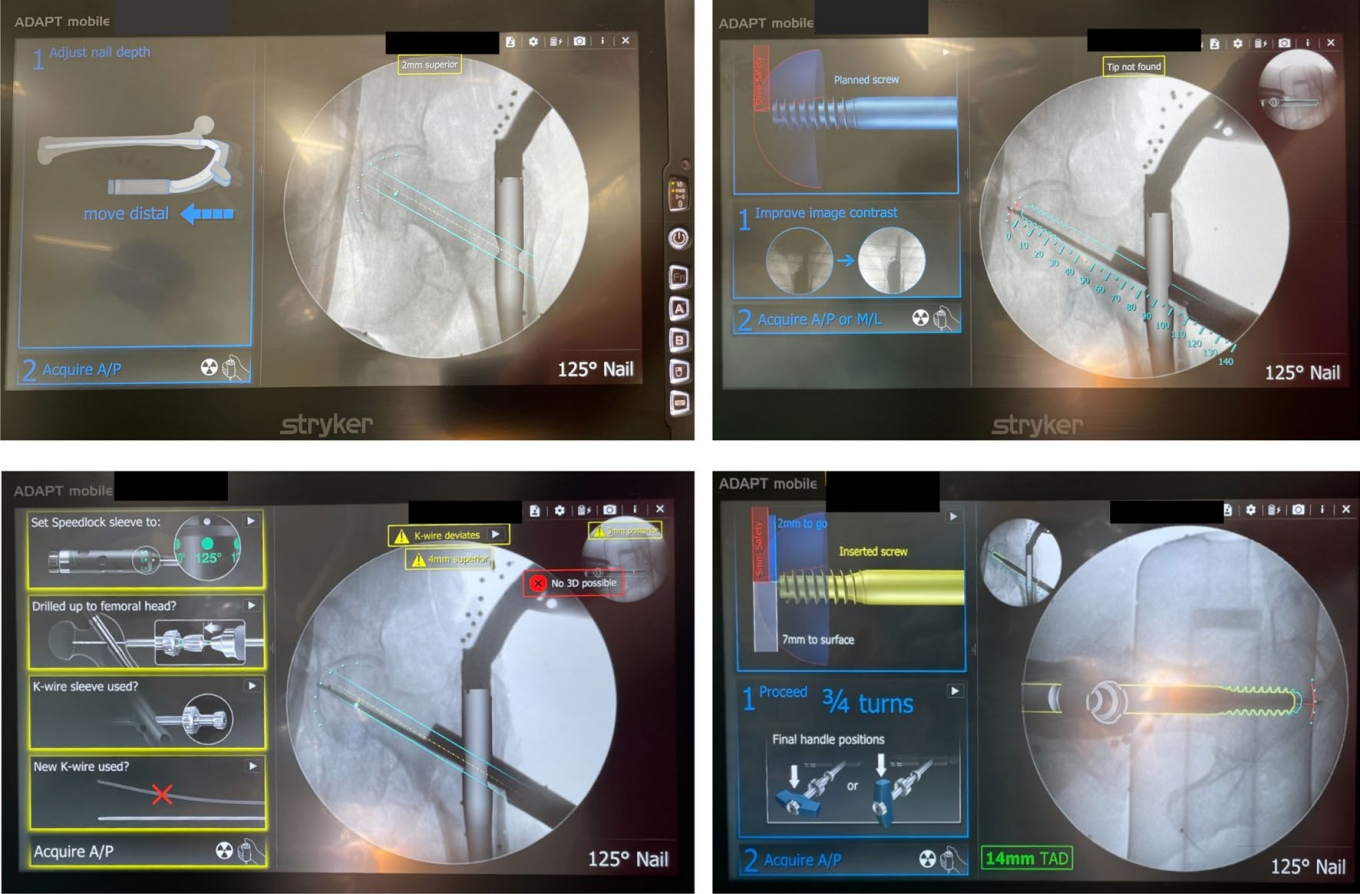
Examples of the intraoperative feedback that ADAPT provides to the surgeon regarding optimization of implant positioning: “Adjust nail depth: move distal” (upper left panel), measurement of the lag screw (upper right panel), “Insert screw: 2 mm to go; proceed ¾ turns” (lower right panel). Several error messages are also displayed “Tip not found” (upper right panel), “No 3D possible” and “Acquire A/P”, i.e., additional fluoroscopy (lower left panel)

The ADAaptive Position Technology (ADAPT) is a computer-assisted navigation system developed by the manufacturer of the Gamma3 IMN (Stryker Trauma GmbH Schönkirchen, Germany) [14]. ADAPT is used intraoperatively with conventional fluoroscopy. ADAPT provides a virtual three-dimensional reconstruction of the proximal femoral anatomy as well as the implant and gives real-time feedback to the surgeon, thereby guiding implant placement based on TAD, Tip-Surface distance (TSD) and centering of the lag screw in the femoral neck. Figure 1 depicts some of the intraoperative feedback the system provides to the surgeon.

Several studies of the ADAPT system have found that ADAPT had a positive effect on the TAD, decreased surgical procedure time, and reduced the use of fluoroscopy [2, 15–21]. However, the perceived usability and acceptability of the ADAPT system have not yet been evaluated. The aim of this study was to investigate the perceived usability and acceptability of the ADAPT system utilizing the System Usability Score (SUS) as well as secondary outcome measures.

## Materials and methods

Prospective observational study carried out in 50 consecutive IMN surgeries for intertrochanteric hip fractures at the Department of Orthopaedics, Aarhus University Hospital, Denmark from February 2021 to October 2021. Approximately 100 IMN for hip fractures are performed annually at the department. During the study period, 13 residents and 19 consultants were on-call. All physicians on-call regularly perform IMN. The ADAPT system was introduced in the department in February 2021, and a general introduction lecture was held for all surgeons and surgical nurses before implementation. Furthermore, a sale representative presented ADAPT and had the system on display at the surgical ward on multiple occasions, where in depth practical introduction and detailed *Instructions for Use* were provided. Guided by the scientific data, the use of ADAPT was made compulsory when treating intertrochanteric femur fractures with the Gamma3 IMN (short and long) from February 2021 to October 2021 [2, 15–21]. No other IMN were applied. A sale representative or a physician with expertise in the use of ADAPT (ADAPT expert: JDR, OB, PHG) were present in the operating room whenever possible.

Following each Gamma3 IMN, the primary surgeon and supervising surgeon (if present) were asked to answer the *System Usability Scale* questionnaire (SUS). The resulting SUS score from the questionnaires were used to quantify perceived usability and acceptability of ADAPT.

### Primary outcome: System Usability Scale (SUS)

SUS is a standardized 10-item questionnaire scored on a hundred-point scale (Table 1) [22, 23]. SUS has been used in previous studies to evaluate usability of surgical aiding systems [24, 25].

**Table 1:** The System Usability Score (SUS) questionnaire was administered after each ADAPT-assisted surgery. For details regarding the Likert-scale and total SUS score calculation, please refer to the text

1. I think that I would like to use ADAPT frequently
2. I found ADAPT unnecessarily complex
3. I thought ADAPT was easy to use
4. I think that I would need the support of a technical person to be able to use ADAPT
5. I found the various functions in ADAPT were well integrated
6. I thought there was too much inconsistency in ADAPT
7. I would imagine that most people would learn to use ADAPT very quickly
8. I found ADAPT very cumbersome (awkward) to use
9. I felt very confident using ADAPT
10. I needed to learn a lot of things before I could get going with ADAPT

Surgeons answered the 10-item SUS questionnaire, by giving points from one to five (Strongly Disagree: 1 point; Disagree: 2 points; Neutral: 3 points; Agree: 4 points; Strongly Agree: 5 points). The highest SUS score is 100 points, with each question having a maximum weight of 10 point. Odd-numbered questions are in a positive tone and even-numbered questions are in a negative tone. Additionally, surgeons could add free text answers to describe positive and negative effects of ADAPT on usability and their general experience with the ADAPT system (Table S1).

The SUS scores were calculated as described by Lewis [23]: SUS Score = 2.5 (20 + (SUM odd-numbered questions) – (SUM even-numbered questions)).

SUS scores were then interpreted according to Bangor et al. [22], on a numeric scale from 0 to 100 and translated to adjective ratings, describing user experience on a 7-point adjective scale (worst imaginable, awful, poor, ok, good, excellent, best imaginable). User acceptability, defined as “not acceptable”, “marginal” or “acceptable”, was also used to interpret the SUS scores.

### Secondary outcomes

In addition to the SUS questionnaire surgeons rated their agreement with three additional statements using the same 5-point scale: (1: “ADAPT helps me to be more effective”; 2: “ADAPT saves me time when I use it”; 3: “I will continue to use ADAPT, when its use is not mandatory anymore”). Lastly, the surgeons had the option to provide free text comments regarding the most-positive and most-negative aspects of ADAPT.

As per hospital protocol, the surgical nurse documented the surgical procedure time, the fluoroscopy time, as well as the implants used in the electronic patient records. Likewise, the anesthesiologist documented the American Society of Anesthesiologists’ physical status classification system (ASA class). The type of intertrochanteric fracture was determined by co-authors R.A.H. and C.L.N.

## Results

Fifty consecutive ADAPT-assisted IMN procedures fractures were performed during the study period. Patient demographics and details are given in Table 2.

**Table 2:** Patient demographics and fracture classification

Patient demographics	ADAPT-assisted femoral nails (*n = *50)
Mean age (95% CI; range)	79.3 (76–83; 45–97)
Sex: male/female	14 / 36
Mean ASA class (95% CI; range)	2.6 (2.5–2.8; 1–4)
Type of nail: short / long	27 / 23
AO/OTA fracture classification: 31A	
31A1.1	1
31A1.2	4
31A1.3	11
31A2.1	6
31A2.2	11
31A2.3	7
31A3.1	3
31A3.2	3
31A3.3	4

*ASA* American Society of Anesthesiologists’ physical status classification system, *AO/OTA* AO Foundation/Orthopaedic Trauma Association

A total of 34 surgeons (17 residents and 17 consultants) used ADAPT at least once as primary surgeons. Residents were the primary surgeon in 37 cases, thereof, 29 were supervised by a more experienced colleague and in 13 of these cases an ADAPT expert (JDR, OB, PHG) was present, while a sales representative was present in 4 cases. Moreover, in 4 of the 13 cases operated by a consultant an ADAPT expert was present. The mean TAD was 16.6 (95% CI 15–18; range: 9–30), mean operation time was 78.7 min (95% CI 69–89; range: 23–211) and the mean fluoroscopy time was 268 s (95% CI 232–303; range: 112–532).

### Primary outcome: SUS

In total, 29 surgeons (16 residents and 8 consultants) completed 61 SUS questionnaires with a median of 2 (range: 1–6) returned SUS questionnaires per surgeon. The overall response was 71% with 78% for primary surgeons and 59% for supervisors. At least 1 SUS questionnaire was returned for 48/50 procedures. The two ADAPT-assisted procedures with missing SUS data were performed by a 3rd and 4th year resident supervised by two different consultants.

**Fig. 2:**
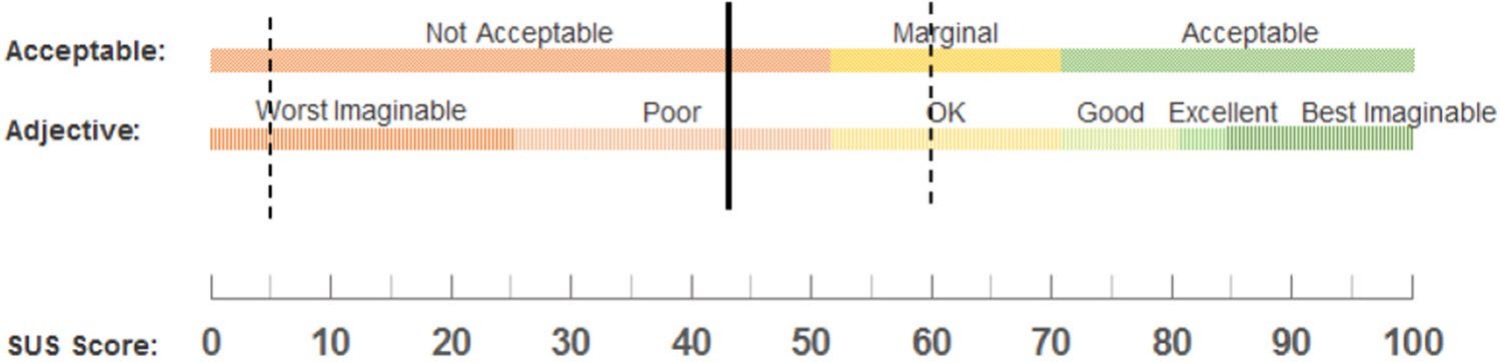
Interpretation of the median SUS score of 43 (range: 5–60) in terms of acceptability and usability [26]. Notably, the most favorable surgeon (outlier) rated ADAPT as SUS = 60, meaning “marginal acceptable”

The median SUS for first-time users rated by all 29 surgeons was 43 (range: 5–60), which translates to “poor” on the adjective rating scale. The median SUS at the latest rated procedure, for those eight surgeons who used the ADAPT three or more times was 38 (range: 21–59), which meant it remained “poor” on the adjective rating scale. There was no statistically significant difference in their first to latest SUS (median difference: 4.3, *p = *0.55). Regarding acceptability the median SUS score means “not acceptable” (Fig. 2). More details regarding residents’ and consultants’ SUS scores as well as the full version of the administered questionnaire is provided as supplementary material.

### Secondary outcomes

**Fig. 3:**
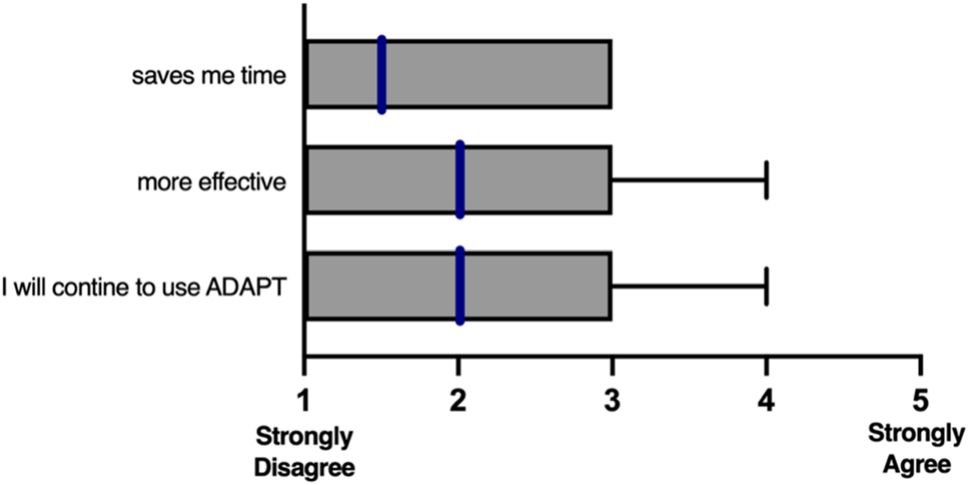
A 5-point Likert scale with anchors: 1—Strongly Disagree and 5—Strongly Agree depicting the response of 29 surgeons with a median 1.5 (range 1–3) for “It saves me time when I use it”; median 2.0 (range 1–4) for “ADAPT helps me to be more effective”; and median 2.0 (range 1–4) for “I will continue to use ADAPT, when its use is not mandatory anymore”. Median values are blue, interquartile range (grey box) and range, i.e., max. value (whiskers)

Regarding the three additional statements, the majority of the 29 surgeons did neither experience that ADAPT saved them time, nor did it help to be more effective (Fig. 3).

Only 5 out of 29 surgeons answered “agree” to the statement “I will continue to use ADAPT, when its use is not mandatory anymore” (3 1st/2nd-year residents and 2 consultants) and none of the surgeons answered “strongly agree”.

Table 3 presents the most positive and most negative aspects of ADAPT based on the free text comments of the collected 61 questionnaires.

**Table 3:** Free-text responses regarding the most-positive and most-negative aspects of ADAPT

Most-positive aspects	Most-negative aspects
“Good visualization of K-wire”	“It complicates instead of assists”
“Accurate prediction of lag screw length”	“Longer surgical procedure time”
“Fewer attempts when placing the K-wire”	“Increased radiation dose due to more intraoperative fluoroscopy”
“Potentially useful for inexperienced surgeons”	“Too inconsistent and too slow image-processing”

## Discussion

In this prospective observational study, we used a qualitative approach to investigate the surgeon’s perceived usability and acceptability of the ADAPT computer-assisted navigation system for intramedullary nailing of hip fractures. We applied the validated SUS questionnaire as primary outcome. During the study period, 29 surgeons performed 50 IMN procedures for intertrochanteric femoral fractures. Based on 61 responses from primary and supervising surgeons, the median SUS score was 43 (range 5–60), meaning “poor” usability and “not acceptable”.

A cadaveric study by Regling et al. investigated lag screw positioning of experienced surgeons and less experienced surgeons with and without ADAPT [27]. They concluded that “both the experienced and less experienced surgeons can benefit from the ADAPT system”. They also showed decrease in fluoroscopy use and no significant change surgical procedure time. Limitations of their study include that only cadaveric unfractured bones were used. As some of the surgeons in our study were 1st year residents, they had little experience with performing IMN procedures. Performing a new procedure and using a new surgical add-on system could have made the task more complex, and thereby potentially amplified a negative effect on the perceived usability and acceptability of ADAPT. On the other hand, computer-assisted navigation systems may provide clear instructions to inexperienced surgeons and thus providing step-by-step guidance during the procedure. This may facilitate learning and give inexperienced surgeons a deeper understanding of the optimal surgical technique, as some users also mentioned in the free text field.

Furthermore, in our study residents and supervisors could prepare, talk together during the procedure, and evaluate the procedure after the surgery and before answering the SUS questionnaire. Supervisors often have more surgical experience and might not see the need for assistance in implant placement, thus rendering the ADAPT system as needless and unnecessary. Therefore, the residents’ answers to our questionnaires could have been negatively biased by the experience and opinion of their supervisor. However, SUS was administered electronically to the surgeons and the surgeons provided their feedback individually.

Despite only eight surgeons using ADAPT 3 or more times, we did not find a trend towards better usability with more exposure to ADAPT. The median SUS of the latest procedure of these eight surgeons was 38, which did not change adjective rating from “poor”. Interestingly, six of the eight primary surgeons who used ADAPT more than three times were residents, i.e., rather novice surgeons who theoretically should benefit most. Moreover, residents are younger and thus more likely to adapt well to new technology.

Most of the surgeons described ADAPT as “unnecessary complicated” and experienced delay and slower progression in the hip fracture procedure when using ADAPT. In some cases, this was due to technical difficulties. The additional screen providing intraoperative guidance and the ADAPT software were described as “slow and inconsistent, in processing fluoroscopic images from the C-arm”. Thereby surgeons answered that ADAPT was “unnecessarily time consuming”, and “increasing fluoroscopy use”. Contrary to our findings, Murakami et al. report significantly shorter surgical procedure time (*p < *0.05) and reduced intraoperative fluoroscopy (*p < *0.01) when using ADAPT (*n = *20) compared without ADAPT (*n = *20) [16]. This is remarkable, but may partly be explained by the fact that a trained computer operator was present for all ADAPT-procedures. Based on their comparative study, they concluded that ADAPT was “a very useful device, for intramedullary nailing of femoral trochanteric fractures”. The perceived usability and acceptability of ADAPT by the surgeons were not reported. Their study as well as our findings highlight that ADAPT is not a plug-and-play device, but training and expert guidance is needed to operate and troubleshoot the system. This is also in line with a recent paper about the effect of new technology on the operating team [28].

In our study, the surgical procedure time in our study was 78.7 min (95% CI 69–89; range: 23–211) and nearly all 29 surgeons marked “strongly disagree” or “disagree” to the statements “it saves me time” and “it makes me effective”. These findings are consistent with Lilly et al. reporting longer surgical procedure time among three fellowship-trained traumatologists when using ADAPT in 50 patients [21]. Notably, only class 31-A1/A2 intertrochanteric femoral fractures were included in the study.

On the contrary, a longitudinal matched cohort study by Herzog et al. found no statistical difference in operating time, comparing periods with and without ADAPT using standardized nail length [2]. It should be noted that none of the studies nor our study report the additional preparation time for setting up ADAPT pre-, and postoperatively.

### Potential educational benefit

Several consultants in our study reported that ADAPT might have a potential educational benefit for younger inexperienced surgeons (Table 3). According to a study by Bjorgul et al. time to completion of a task and use of fluoroscopy are representable indicators of surgical performance when performing hip fracture surgery [29]. Therefore, if ADAPT have educational benefits it should improve the TAD, surgical procedure time, and decrease the use of fluoroscopy. Several previous studies have shown improved TAD when using ADAPT, but to evaluate the educational benefit of ADAPT future studies must compare standard surgery results after a period of ADAPT use [2, 15–21, 30].

### Limitations

To our knowledge, System Usability Scale have been used for evaluation of surgical aids/devices but has not been formally validated for this purpose. A second limitation is that 29 of 50 procedures were performed by a resident, with a more experienced surgeon supervising the procedure, who may not see the need for computer-assisted navigation. Interestingly, it was the consultants who reported a potential benefit for more inexperienced users, while this was not the case for the actual stakeholder, i.e., the residents. A third limitation is that it was mandatory to use ADAPT in the study period, therefore, some surgeons were forced to use ADAPT and thus be subjected to change, which may have negatively influenced their perception of the new technology. We made it compulsory to use ADAPT based on the current body of evidence and in order to resurrect it’s use as it had been available for surgeons in our department for more than a year before the study period, but had rarely been used.

Theoretically, it would be of interest to compare SUS scores of Gamma 3 nailing with and without the ADAPT system. However, SUS is specifically designed to evaluate the usability of computer systems and technical aids. It is not designed, nor has it been used to evaluate specific implants or surgical techniques that do not include computer assistance and navigation.

Finally, an individual SUS-responses by a surgeon may be considered subjective. However, an argument can be made that collecting multiple responses from many surgeons provides an objective assessment of the system’s usability (in the present manuscript 61 SUS from 29 surgeons). In a similar fashion, this argument also applies for the “Objective Structured Assessment of Technical Skills (OSATS)”, which is widely used in education. Individual responses are subjective, but a sound and objective evaluation can be made, if multiple assessments are collected and these assessments align. Regarding the present study, we would like to highlight that the spread of evaluations by surgeons and residents at our level 1 trauma center ranged from “not-acceptable” to “marginal acceptable”. Therefore, the individual subjective assessments align and a firm and objective conclusion regarding the usability of the ADAPT system can be drawn.

## Conclusions

The usability and acceptability of ADAPT was “poor” or “not acceptable” based on our prospective observational study evaluating the compulsory use of the system in 50 consecutive hip fracture patients at our level 1 trauma center, by the majority of operating surgeons. Although the majority found it unnecessary and time-consuming, some stated that it might be a useful supplemental tool for inexperienced surgeons. The System Usability Scale may be used in further research exploring usability and acceptability of novel computer-assisted navigation systems for orthopaedic surgery.

### Supplementary Information

Below is the link to the electronic supplementary material.

Supplementary file1 (PDF 80 KB)

## Data Availability

Data may be requested from the corresponding author.
