# Supplementary material for: Poor usability of computer-assisted navigation for hip fracture surgery
Source: Arch Orthop Trauma Surg. 2023 Oct 25;144(1):251–7. doi: 10.1007/s00402-023-05096-2 (PMC10774189; doi:10.1007/s00402-023-05096-2)
Supplement: Supplementary file 1 — Supplementary file1 (PDF 80 KB) [file 402_2023_5096_MOESM1_ESM.pdf]

regionsid:

____

operation date + approx. time:

____

your role:

☐ primary surgeon
☐ assistant
☐ supervisor

How many times have you used ADAPT (no.)?

____

System Usability Scale

For each of the following statements, please mark on box that best describes your reaction to the tool ***ADAPT***.

	NOT APPLICABLE	1 - Strongly Disagree	2	3	4	5 - Strongly Agree
1. I think that I would like to use ADAPT frequently.	☐	☐	☐	☐	☐	☐
2. I found ADAPT unnecessarily complex.	☐	☐	☐	☐	☐	☐
3. I thought ADAPT was easy to use.	☐	☐	☐	☐	☐	☐
4. I think that I would need the support of a technical person to be able to use ADAPT.	☐	☐	☐	☐	☐	☐
5. I found the various functions in ADAPT were well integrated.	☐	☐	☐	☐	☐	☐
6. I thought there was too much inconsistency in ADAPT.	☐	☐	☐	☐	☐	☐
7. I would imagine that most people would learn to use ADAPT very quickly.	☐	☐	☐	☐	☐	☐
8. I found ADAPT very cumbersome (awkward) to use.	☐	☐	☐	☐	☐	☐
9. I felt very confident using ADAPT.	☐	☐	☐	☐	☐	☐
10. I needed to learn a lot of things before I could get going with ADAPT.	☐	☐	☐	☐	☐	☐
11. ADAPT helps me to be more effective	☐	☐	☐	☐	☐	☐
12. It saves me time when I use it	☐	☐	☐	☐	☐	☐
13. I will continue to use ADAPT, when its use is not mandatory anymore?	☐	☐	☐	☐	☐	☐

List the most positive aspect(s) of ADAPT:

List the most negative aspect(s) of ADAPT:
